# Sleep and Oxidative Stress: Current Perspectives on the Role of NRF2

**DOI:** 10.1007/s10571-024-01487-0

**Published:** 2024-06-25

**Authors:** Sergio Davinelli, Alessandro Medoro, Rosa Savino, Giovanni Scapagnini

**Affiliations:** 1 Department of Medicine and Health Sciences “V. Tiberio”, University of Molise, Via F. De Sanctis, s.n.c., 86100 Campobasso, Italy; 2 Department of Woman and Child, Neuropsychiatry for Child and Adolescent Unit, General Hospital “Riuniti” of Foggia, Viale Pinto Luigi, 1, 71122 Foggia, Italy

**Keywords:** Sleep, Sleep disturbances, NRF2, Oxidative stress, Antioxidants

## Abstract

Sleep is a fundamental conserved physiological state across evolution, suggesting vital biological functions that are yet to be fully clarified. However, our understanding of the neural and molecular basis of sleep regulation has increased rapidly in recent years. Among various processes implicated in controlling sleep homeostasis, a bidirectional relationship between sleep and oxidative stress has recently emerged. One proposed function of sleep may be the mitigation of oxidative stress in both brain and peripheral tissues, contributing to the clearance of reactive species that accumulate during wakefulness. Conversely, reactive species, such as reactive oxygen species (ROS) and reactive nitrogen species (RNS), at physiological levels, may act as signaling agents to regulate redox-sensitive transcriptional factors, enzymes, and other effectors involved in the regulation of sleep. As a primary sensor of intracellular oxidation, the transcription factor NRF2 is emerging as an indispensable component to maintain cellular redox homeostasis during sleep. Indeed, a number of studies have revealed an association between NRF2 dysfunction and the most common sleep conditions, including sleep loss, obstructive sleep apnea, and circadian sleep disturbances. This review examines the evidence of the intricate link between oxidative stress and NRF2 function in the context of sleep, and highlights the potential of NRF2 modulators to alleviate sleep disturbances.

**Graphical Abstract:**

A bidirectional relationship between sleep and oxidative stress has been shown, indicating that sleep may play a protective role against the accumulation of reactive species during wakefulness and sleep deprivation. However, reactive species might also serve as signaling molecules that influence sleep regulation mechanisms. Notably, as a sensor of cellular redox changes, the transcription factor NRF2 is emerging as a key regulator of sleep homeostasis.

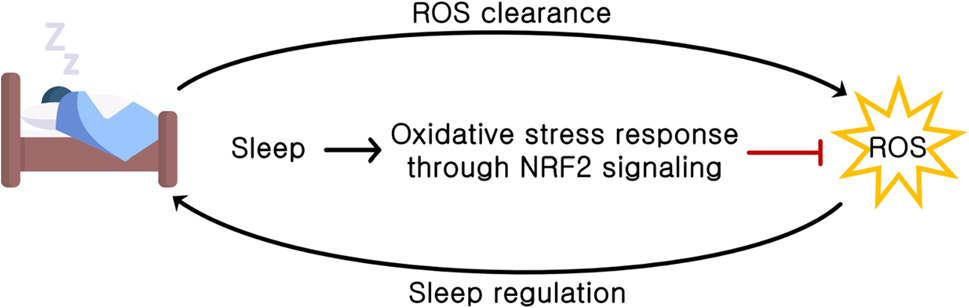

## Introduction

Although it may manifest in various forms, sleep is an evolutionarily conserved behavior across the animal kingdom, highlighting its ancient and significant role throughout the phylogenetic tree (Joiner 2016). In humans, sleep is fundamental to brain function and systemic physiology, affecting metabolism, the regulation of appetite, and the functionality of the immune, endocrine, and cardiovascular systems. Collectively, deficiencies in the quantity and quality of sleep, known as sleep disturbances, can interfere with sleep continuity and are associated with significant adverse health outcomes in both the short and long term (Medic et al. 2017). Poor sleep is consistently associated with the development of numerous pathological conditions, such as neurodegenerative disorders, depression, cardiovascular and metabolic diseases, and various forms of cancer (Pillai and Leverenz 2017; Cappuccio and Miller 2017; Freeman et al. 2020; Song et al. 2021). The regulation of sleep patterns and needs is influenced by multiple factors, including chronological age, developmental stage, genetics, behavior, environmental variables, and social influences (Grandner 2017; Nollet et al. 2023). Furthermore, sleep regulation is also governed by the circadian timing system, specialized neural circuits dedicated to the homeostatic control of sleep, and the neuroendocrine system (Yin et al. 2017; Smith and Mong 2019; Bacaro et al. 2020; Pandi-Perumal et al. 2022). There is substantial epidemiological evidence suggesting that adults require an average sleep duration of 7–8 h per night for optimal health. Indeed, both short and long sleep duration are associated with an increased risk of all-cause mortality and adverse health outcomes, showing a *U*-shaped relationship (Chaput et al. 2020).

Sleep disturbances have a negative impact on the signaling pathways that regulate structural and synaptic plasticity, such as those involving cyclic adenosine monophosphate (cAMP), glutamatergic signaling, mammalian target of rapamycin (mTOR), protein synthesis, and transcriptional mechanisms. This can lead to disruptions in synaptic integrity and neural circuitry (Cirelli 2002; Vecsey et al. 2009; Tudor et al. 2016; Briggs et al. 2018). Sleep abnormalities have also been linked to peripheral inflammation and immune dysfunction. This association is evidenced by elevated serum levels of pro-inflammatory cytokines, such as interleukin-1 (IL-1), tumor necrosis factor α (TNF-α), IL-6, and IL-17. Additionally, sleep disturbances can lead to altered activity of immune cells, including macrophages and natural killer cells. Activation of peripheral immune cells and systemic inflammation may contribute to neuroinflammation, leading to the activation of microglia and astrocytes with the subsequent release of inflammatory and signaling molecules. These include cytokines, chemokines, prostaglandins, nitric oxide (NO), cyclooxygenase-2 (COX-2), and reactive oxygen species (ROS) (Hurtado-Alvarado et al. 2018; Morris et al. 2018; Manchanda et al. 2018). Although the precise pathophysiological mechanisms underlying sleep abnormalities remain elusive, sleep disruption is often accompanied by an increase in radical species, such as ROS and reactive nitrogen species (RNS). This increase contributes to a pathophysiological loop where immune activation, (neuro)inflammation, and oxidative stress interact reciprocally, potentially exacerbating sleep abnormalities (Morris et al. 2018; Xue et al. 2019).

Oxidative stress occurs when the production of ROS or RNS exceeds the antioxidant capacity of the cells, resulting in structural damage and oxidative modifications to proteins, lipids, and nucleic acids. Although the relationship between sleep and oxidative stress is still under intense investigation, several studies provide evidence that wakefulness represents an oxidative challenge for the brain (D’Almeida et al. 1998; Everson et al. 2005; Hill et al. 2018).

Sleep–wake cycles influence metabolic rate and energy metabolism, leading to increased mitochondrial ROS production during wakefulness through enhanced oxidative phosphorylation, and also impact the production of RNS via different NO synthases, crucial for regulating sleep and maintaining homeostasis (Cespuglio et al. 2012; Hartmann and Kempf 2023). However, the relationship between sleep and oxidative stress remains unclear and several groups published contradictory findigs. The free radical flux theory of sleep, proposed by Reimund in 1994, is a controversial hypothesis that seeks to explain one of the purposes of sleep. This hypothesis suggests that sleep may allow the elimination of free radicals accumulated in the brain during wakefulness (Reimund 1994). Moreover, it has been hypothesized that, during sleep, the oxidative detoxification of the brain may be facilitated by uridine and glutathione (GSH), which potentiate GABAergic transmission and inhibit glutamatergic transmission, respectively (Inoué et al. 1995). In studies involving sleep-deprived rats, findings revealed reduced levels of glutathione, superoxide dismutase (SOD), and catalase (CAT) in both brain and liver tissues (D’Almeida et al. 1998; Ramanathan et al. 2002; Everson et al. 2005). Conversely, other data indicate no significant change in antioxidant activity and no evidence of oxidative damage in the brains of sleep-deprived rats (D’Almeida et al. 1997; Gopalakrishnan et al. 2004). Although the majority of evidence originates from animal studies, two recent systematic reviews have attempted to elucidate the relationship between sleep and oxidative stress. These reviews highlight the antioxidant effects of sleep in both brain and peripheral tissues, as well as the occurrence of oxidative stress following sleep deprivation (Villafuerte et al. 2015; Neculicioiu et al. 2023).

The transcription factor nuclear factor erythroid 2-related factor 2 (NRF2) is recognized as a master regulator of the cellular antioxidant response. The function of NRF2 is ubiquitously conserved across different cell types, but it is widely expressed in the central nervous system (CNS), where it regulates a range of functions including redox balance, xenobiotic metabolism, inflammation, and proteostasis (Cuadrado 2022). NRF2 plays a role in regulating the expression of many genes, most of which are responsible for producing antioxidant and detoxification enzymes. These enzymes alleviate cell damage and maintain redox homeostasis. Moreover, NRF2 contributes to the anti-inflammatory process by inhibiting the release of pro-inflammatory genes, thus attenuating the pro-inflammatory phenotype that characterizes numerous pathophysiological conditions. (Malhotra et al. 2010; Saha et al. 2020). Due to its numerous functions, NRF2 has recently become an attractive target of research in sleep disturbances and disorders. This review discusses the role of NRF2 in the context of oxidative stress linked to sleep abnormalities, along with the current advancements in nutritional and pharmacological activators that may modulate its activity during sleep.

## NRF2 Pathway: Mechanisms and Functions

NRF2 is a transcription factor that belongs to the cap 'n' collar (CnC) family and contains a basic-region leucine zipper (bZIP) domain. It plays a crucial role in regulating numerous antioxidant genes and drug-metabolizing enzymes. In the nucleus, NRF2 forms a heterodimer with other bZip proteins, such as small avian musculoaponeurotic fibrosarcomas (MAFs) F, G, and K. Together, they bind to specific regions of DNA called antioxidant response elements (AREs), which are located in the promoter or enhancer regions of NRF2 target genes. The regulatory elements within the ARE sequences of these genes confer specificity to the NRF2 transcriptional signature, which varies according to cell type, intensity, and duration of the stimuli (Lee et al. 2005; Bai et al. 2019; Liu et al. 2019). As mentioned, in the brain, these genes regulate a variety of biological processes involved in the physiology of sleep and its disturbances (Sandberg et al. 2014; Zhou et al. 2018; Hafycz and Naidoo 2019; Morrone et al. 2023).

The activity of NRF2 is primarily regulated post-translationally through its interaction with Kelch-like ECH-associated protein 1 (KEAP1), a crucial regulator of NRF2 under basal redox conditions. During cellular homeostasis, KEAP1 binds to NRF2 in the cytoplasm, leading to its rapid degradation through ubiquitination by the Cullin 3(Cul3)/ring-box 1 (RBX1) complex. KEAP1 also functions as a sensor for oxidative stress. Upon oxidative stress, specific cysteine residues in KEAP1 are modified, causing conformational changes that disrupt the KEAP1-NRF2 interaction. This disruption prevents KEAP1 from binding to newly synthesized NRF2, allowing NRF2 to accumulate in the nucleus and activate genes regulated by AREs. (Suzuki and Yamamoto 2017; Suzuki et al. 2023). While NRF2 is predominantly regulated via its interaction with KEAP1, emerging evidence indicates that a variety of KEAP1-independent mechanisms also modulate NRF2 activity. These mechanisms act under both redox-sensitive and -insensitive conditions, influencing NRF2 at the transcriptional, post-transcriptional, and post-translational levels, and involving a range of proteins and epigenetic factors such as DNA methylation, histone modifications, and microRNA (Bryan et al. 2013; Cheng et al. 2016; Mahajan and Sitasawad 2021). Additionally, cell signaling pathways contribute to NRF2 regulation, highlighting the complex regulatory network underlying NRF2 activity. For example, in a resting state, glycogen synthase kinase-3 (GSK-3) phosphorylates NRF2, creating a site recognized by the E3 ligase adapter beta-transducin repeat-containing protein (β-TrCP). This recognition, mediated by the Cul3/RBX1 complex, leads to the ubiquitination of NRF2 and its subsequent degradation via the proteasome (Cuadrado 2015).

As previously mentioned, NRF2 plays a crucial role in regulating various antioxidant enzymes that neutralize harmful reactive oxidants and electrophiles, converting them into less harmful forms (Table 1). This regulatory function is essential for maintaining redox balance and protecting against oxidative damage. Classical enzymes controlled by NRF2, such as SOD, CAT, and heme oxygenase-1 (HO-1), are localized in various organelles and subcellular locations, where they actively scavenge ROS and neutralize electrophiles (Cuadrado et al. 2019). For example, HO-1, which is sensitive to oxidative stress, contributes to the production of bilirubin, a powerful physiological antioxidant (Drummond et al. 2019). Additionally, NRF2 regulates the expression of enzymes such as glucose 6-phosphate dehydrogenase (G6PD), 6-phosphogluconate dehydrogenase (6PGD), malic enzyme 1 (ME1), and isocitrate dehydrogenase 1 (IDH1), which are involved in generating reduced nicotinamide adenine dinucleotide phosphate (NADPH), a crucial cofactor for antioxidant reactions (Wu et al. 2011). NRF2 also plays a key role in modulating the GSH and thioredoxin (Trx) antioxidant systems, which are important for restoring redox balance. It is noteworthy that NRF2 regulates the expression of enzymes necessary to synthesize GSH, including glutamate-cysteine ligase catalytic subunit (GCLC) and its regulatory subunit (GCLM), glutathione reductase (GR), glutathione peroxidase (GPx), and several glutathione S-transferases (GST) (Suh et al. 2004).

**Table 1:** NRF2-dependent antioxidant genes and their enzymatic functions as discussed in this review

NRF2-dependent genes	Enzymatic function
Superoxide dismutase (SOD)	Catalyzes the dismutation of the superoxide radical (O_2_^−^) into molecular oxygen (O_2_) and hydrogen peroxide (H_2_O_2_)
Catalase (CAT)	Catalyzes the decomposition of hydrogen peroxide (H_2_O_2_) to water and molecular oxygen (O_2_)
Heme oxygenase 1 (HO-1)	Catalyzes the degradation of heme to produce biliverdin and carbon monoxide (CO), two molecules with antioxidant effects
Glucose 6-phosphate dehydrogenase (G6PD)	Catalyzes the rate-limiting step of the oxidative pentose-phosphate pathway and generates nicotinamide adenine dinucleotide phosphate (NADPH)
6-phosphogluconate dehydrogenase (6PGD)	Produces NADPH by converting 6-phospho D-gluconolactone to D-ribulose 5-phosphate in the pentose-phosphate pathways
Malic enzyme 1 (ME1)	Catalyzes the conversion of malate to pyruvate while concomitantly generating NADPH from NADP
Isocitrate dehydrogenase 1 (IDH1)	Catalyzes decarboxylation of isocitrate to produce NADPH and 2-ketoglutarate
Thioredoxin (Trx) antioxidant system	Protects against oxidative stress through its disulfide reductase activity regulating protein dithiol/disulfide balance
Glutamate-cysteine ligase catalytic subunit (GCLC) and modifier subunit (GCLM)	Encode the subunits of the enzyme glutamate-cysteine ligase (GCL), which is the rate-limiting enzyme for the synthesis of the antioxidant glutathione (GSH)
Glutathione reductase (GR)	Catalyzes reduction of oxidized GSH and regeneration of reduced GSH
Glutathione peroxidase (GPx)	Reduces lipid hydroperoxides to their corresponding alcohols and H_2_O_2_ to water
Glutathione S-transferases (GST)	Catalyzes the conjugation of various substrates to GSH detoxifying endogenous compounds and enabling the breakdown of xenobiotics
NAD(P)H quinone oxidoreductase 1 (NQO1)	Flavin-containing quinone reductase that catalyzes two-electron reduction of quinones to hydroquinones
Peroxiredoxins (Prdx)	Regulate peroxide levels within cells, including H_2_O_2_ and peroxynitrite (ONOO^−^)

Nevertheless, NRF2 orchestrates the regulation of an extensive array of genes, encompassing not only various families of antioxidant enzymes but also those involved in non-cytochrome P450 phase-I and phase-II drug metabolism. Additionally, it modulates genes responsible for the regulation of uptake and efflux transporters, influencing the kinetics and disposition of xenobiotics (Anwar-Mohamed et al. 2011; Wu et al. 2012). It has also been reported a relevant role of NRF2 in the resolution of inflammation through several mechanisms inhibiting the expression of inflammatory mediators. Recent studies highlight the synergistic interaction between NRF2 and nuclear factor-κB (NF-κB) signaling pathways in maintaining cellular redox homeostasis and modulating responses to oxidative stress and inflammation (Wardyn et al. 2015; Davinelli et al. 2022). The enhanced expression of HO-1, driven by NRF2, leads to the suppression of NF-κB, thereby reducing the production of inflammatory mediators. This activation of the NRF2/HO-1 axis is often linked with a decrease in NF-κB activity (de Oliveira et al. 2018; Gu et al. 2020). Enzymes regulated by NRF2, such as NAD(P)H quinone oxidoreductase 1 (NQO1), GCLC, and HO-1, have been found to suppress various cytokines and chemokines, including TNF-α, IL-6, IL-1β, and monocyte chemoattractant protein-1 (MCP-1) (Ahmed et al. 2017). Interestingly, NRF2 is able to inhibit the activation of inflammatory genes without binding to the ARE sequence. Indeed, NRF2 can bind  to the promoter regions of pro-inflammatory cytokines (e.g., IL-6 and IL-1β), preventing the recruitment of RNA polymerase II and thus inhibiting their gene transcription (Kobayashi et al. 2016).

## Sleep and Oxidative Stress

Sleep is thought to be regulated by circadian and homeostatic processes. The circadian clock controls the timing of sleep, regulating the sleep–wake cycle and the distribution of sleep stages (i.e., rapid eye movement [REM] and non-rapid eye movement [NREM]) throughout the night. This system also exerts a pivotal influence on energy metabolism, functioning at both behavioral and molecular levels. It modulates aspects such as feeding behavior, mood, and alertness, while also regulating molecular pathways involved in lipid and glucose metabolism and inflammatory responses. Conversely, the mechanisms of sleep homeostasis are less defined but it is hypothesized that they play a role by monitoring variables that accumulate during wakefulness and triggering sleep once a certain threshold is reached (Laposky et al. 2008; Borbély et al. 2016; Deboer 2018). Therefore, it is unsurprising that inadequate sleep (either too long or too short) may disrupt the delicate balance between the circadian and homeostatic sleep regulatory mechanisms, leading to detrimental physiological consequences (Grandner et al. 2012; Nassan and Videnovic 2022; Lane et al. 2023).

Recent research has begun to reveal the molecular mechanisms by which redox signaling influences sleep–wake patterns. Evidence suggests that the cellular redox state plays a role in regulating neuronal activity and clock gene transcription in the suprachiasmatic nucleus (SCN). ROS produced by activated microglia and astrocytes may disrupt the function of the master clock, leading to circadian rhythm disturbances (Rutter et al. 2001; Lananna et al. 2018). Oxidative stress can also impact signaling pathways such as adenosine monophosphate kinase (AMPK) and the peroxiredoxin (Prdx) system, potentially compromising cellular antioxidant defenses controlled by NRF2 (Lee and Kim 2013; Hoyle and O’Neill 2015; Petsouki et al. 2022; Mezhnina et al. 2022). Noteworthy, AMPK acts as a key metabolic sensor that reacts to changes in sleep/wake states and cellular energy, regulating sleep homeostasis (Chikahisa et al. 2009; Dworak et al. 2010). Animal studies have shown that sleep disruption itself can increase oxidative stress levels, highlighting the complex interplay between redox signaling and sleep regulation (Villafuerte et al. 2015). Consequently, ROS levels likely increase during prolonged wakefulness, suggesting that sleep may function in ROS clearance or that sleep deprivation exacerbates ROS accumulation. Despite the controversies surrounding the role of ROS in sleep homeostasis, recent findings indicate that sleep deprivation modifies the redox balance within key neurons that regulate sleep in the fly brain, thereby impacting their functional activity (Kempf et al. 2019). Other studies have revealed signs of oxidative stress without detecting oxidative damage. Conversely, some studies have observed oxidative modification of lipids and DNA, while others have found no significant changes (D’Almeida et al. 1997; Gopalakrishnan et al. 2004; Andersen et al. 2009; Gulec et al. 2012; Lima et al. 2014; Hill et al. 2018; Vaccaro et al. 2020). Methodological aspects, such as differences in sleep deprivation protocols, brain regions analyzed, and experimental models, along with the observation that the brain does not appear to be significantly damaged by oxidative stress after sleep deprivation, have led to search for signs of oxidative stress in other organs. Several studies show that gut, lung, and liver are prone to significant oxidative damage after sleep deprivation. Using flies and mice, it has been demonstrated that sleep deprivation results in the accumulation of ROS with consequent oxidative stress in the gut, rather than in the brain (Vaccaro et al. 2020). This is consistent with other findings showing a weakening in the antioxidant defences and oxidative DNA damage in the gut, liver, spleen, and lung of animals after sleep restriction. (Everson et al. 2005, 2014; Lungato et al. 2013; Villafuerte et al. 2015; Pandey and Kar 2018). In humans, night workers have been observed to exhibit elevated systemic markers of oxidative stress (e.g., plasma protein oxidation) and reduced antioxidant defenses (e.g., CAT, SOD, and GPx) when compared to their day worker counterparts (Teixeira et al. 2019). Given that one proposed function of sleep is the prevention of oxidative stress, it is plausible that sleep plays a role in the repair and homeostasis of peripheral tissues. Additionally, as ROS are increasingly recognized as critical signaling molecules in the homeostatic control of various processes, they could act as signals of cellular stress from peripheral tissues, influencing the mechanisms of sleep regulation and initiation (Ikeda et al. 2005; Anafi et al. 2013; Sies and Jones 2020). Oxidative stress has been identified as a key regulator of sleep homeostasis, influencing various mechanisms (Neculicioiu et al. 2023; Terzi et al. 2024). Loss of sleep has been shown to elevate levels of ROS, leading to the activation of different response pathways. Sleep deprivation can disrupt oxidative phosphorylation, resulting in a decoupling of ATP generation. Additionally, sleep deprivation has been linked to the induction of the endoplasmic reticulum (ER) unfolded protein response (UPR). The association between sleep deprivation and the activation of the UPR in the ER has been observed in both fly heads and mouse brains. The UPR is a signal transduction pathway that senses the fidelity of protein folding in the ER lumen (Naidoo et al. 2005; Brown et al. 2017). Other consequences of sleep loss, especially chronic sleep deprivation, include mitochondrial dysfunction, as evidenced by reduced levels of cytochrome C oxidase, mitochondrial membrane potential, and ATP production (Zhao et al. 2016).

**Fig. 1:**
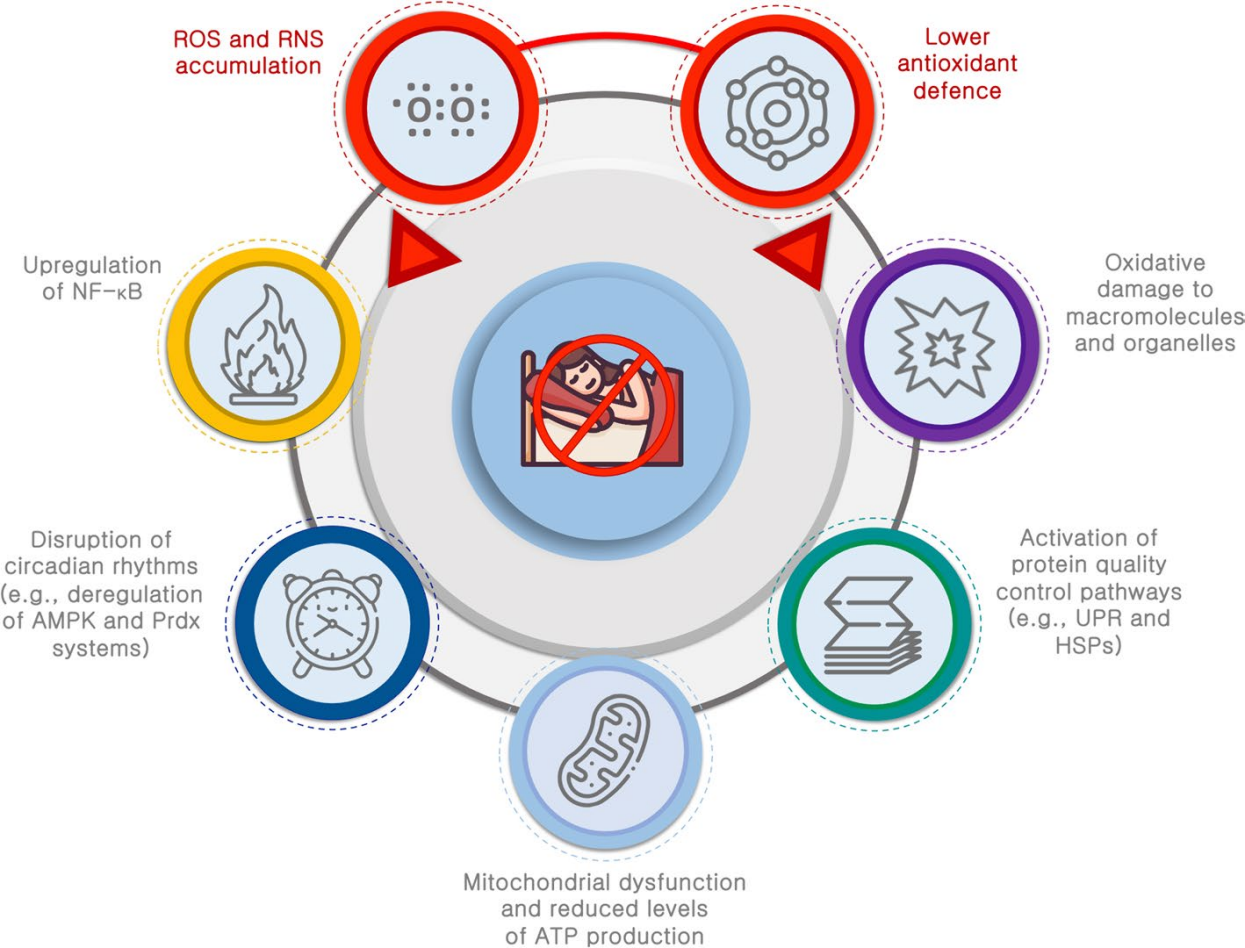
Redox consequences induced by sleep deprivation. Prolonged wakefulness, a common aspect of various sleep disturbances, results in increased levels of reactive oxygen (ROS) and reactive nitrogen (RNS) species, and a reduction in antioxidant defences. Consequently, oxidative/nitrosative stress can lead to oxidative damage and activation of nuclear factor-κB (NF-κB). Oxidative stress also disrupts circadian rhythms by altering adenosine monophosphate kinase (AMPK) signaling pathway and inactivating the peroxiredoxin (Prdx) system. Under sleep deprivation, the increase of the physiological levels of ROS activates different response pathways, such as unfolded protein response (UPR) and heat shock protein (HSP) pathways, to mitigate oxidative damage. Finally, sleep deprivation may impair mitochondrial function and reduce ATP production

The synthesis of heat-shock proteins (HSPs) represents one of the most efficient cellular defense mechanisms. Terao and colleagues have shown that sleep deprivation triggers the upregulation of certain HSP family genes in the brain (Terao et al. 2003). Consequently, the induction of HSPs and the increased expression of uncoupling proteins may serve as neuroprotective strategies to counteract oxidative damage during periods of sleep deprivation. Although inflammation contributes to the development of sleep dyshomeostasis (Terao et al. 1998; Oishi et al. 2015), the production of ROS and RNS also exerts a significant influence on impaired sleep homeostasis. Sleep deprivation elevates the production of NO and RNS, which catalyze the nitrosylation of crucial proteins, including the N-ethylmaleimide sensitive factor (NSF) and postsynaptic density-95 (PSD-95). These modifications impact the function and expression of glutamatergic AMPA and NMDA receptors, consequently deregulating the SCN (Huang et al. 2005; Ho et al. 2011; Neculicioiu et al. 2023). This leads to circadian irregularities and sleep disturbances. Another mechanism involves the oxidative inactivation of cyclic guanosine monophosphate (cGMP), which mediates the effects of NO on sleep homeostasis, and the upregulation of NF-kB by inducible nitric oxide synthase (iNOS) from activated microglia, significantly influencing sleep homeostasis (Cespuglio et al. 2012; Morris et al. 2018). The major redox consequences associated with a lack of adequate sleep are depicted in Fig. 1.

## Role of NRF2 in Sleep Disturbances

**Fig. 2:**
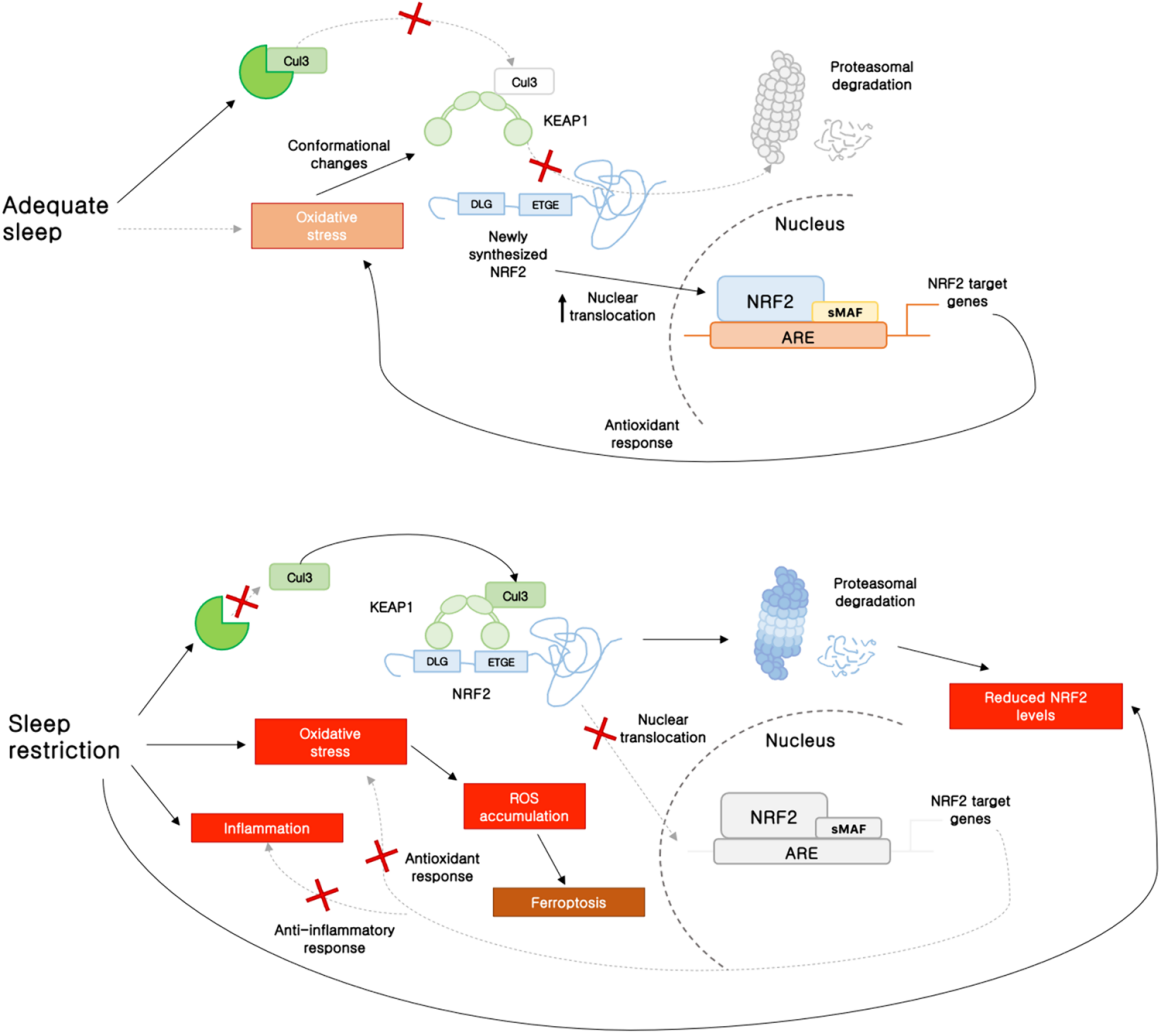
Regulation of the NRF2 signaling pathway during sleep. After adequate sleep, oxidative stress accumulated during wakefulness is cleared through an appropriate response. Notably, oxidative stress can induce a conformational change in the KEAP1–Cul3–E3 ubiquitin ligase complex by modifying specific cysteine residues in KEAP1. These changes disrupt the two-site binding of NRF2 to KEAP1 (specifically, the ETGE and DLG motifs) and reduce Cul3 availability in the NRF2 ubiquitination complex. Consequently, this leads to the translocation and accumulation of NRF2 in the nucleus, where it dimerizes with members of the sMaf protein family and binds to ARE within the regulatory regions of various cell defense genes. Sleep restriction results in increased availability of Cul3 in the NRF2 ubiquitination complex, thereby sequestering NRF2 and preventing its translocation to the nucleus and subsequent activation of antioxidant genes. Therefore, antioxidant response is not appropriately upregulated leading to increased oxidative stress and inflammation. Additionally, the dysregulation of NRF2 and its target genes can influence the propagation of the ferroptotic signal

A growing body of evidence suggests significant alterations in redox balance associated with sleep disturbances, indicating impaired metabolic clearance and increased oxidative stress in the CNS and peripheral tissues (Everson et al. 2005; Trivedi et al. 2017; Hill et al. 2018; Chen et al. 2022b). Inadequate sleep, particularly chronic sleep deprivation, results in a decrease in NRF2 transcriptional activity, leading to an impaired antioxidant response to oxidative stress induced by sleep restriction (Fig. 2). Sleep disturbances are frequently linked to sleep disorders, including obstructive sleep apnea (OSA). Characterized by intermittent hypoxia (IH), OSA is associated with increased ROS and RNS, adversely impacting cardio-/cerebro-vascular conditions in OSA patients. Currently, the NRF2-regulated antioxidant system is emerging as a critical player in the context of OSA, becoming dysregulated due to IH and elevated levels of ROS. This dysregulation can lead to insufficient antioxidative responses, contributing to the progression of OSA-related complications (Lavie 2015). Patients with moderate to severe OSA exhibited reduced expression  of NRF2 and its downstream targets, such as  HO-1, along with  decreased plasma levels of SOD and Trx. This NRF2 dysregulation has been strongly linked to deficits in memory and executive function, indicating its potential role in the cognitive decline observed in these patients (Zhou et al. 2018). In support of a role of NRF2 in OSA, it has also been found that restoration of NRF2 activity can mitigate cognitive impairment and inflammation in a model of IH and sleep fragmentation by modulating ER stress and regulating the expression of the antioxidant gene Prdx1 (Qiu et al. 2023). Additionally, OSA simulated by IH increased serum levels of inflammatory and oxidative markers, such as C-reactive protein (CRP), TNF-α, IL-1β, IL-6, malondialdehyde (MDA), and 8-isoprostane. These increases activated NRF2 and its downstream target HO-1 in a dose-dependent manner to attenuate the inflammation caused by IH in the pulmonary tissue (Wang et al. 2017).

Ferroptosis, an iron-dependent non-apoptotic cell death characterized by accumulation of lipid peroxides and ROS, has been shown to be associated with sleep deprivation and NRF2 pathway. Maternal sleep deprivation-induced microglial activation, lipid peroxidation, and release of inflammatory factors, causing memory impairment and nerve damage in offspring rats characterized by ferroptosis in the hippocampus. Concurrently, this damage has been associated with reduced levels of NRF2 and HO-1 (Lv et al. 2023). Other studies in sleep-deprived animals support the potential role of NRF2 dysfunction during sleep loss. In mice subjected to sleep deprivation, it has been found that activation of microglia and astrocytes was accompanied by downregulation of α7 nicotinic acetylcholine receptor (α 7-nAChR), which is involved in the regulation of calcium-dependent events and cholinergic anti-inflammatory pathway. This change in α 7-nAChR occurred with an increase of pro-inflammatory mediators and reduced levels of NRF2 and HO-1 (Xue et al. 2019). The genetic manipulation of NRF2 signaling positively influences aging, synapse function, and sleep behavior in fly. Indeed, the suppression of KEAP1, the NRF2 inhibitor, enhances survival and synaptic performance, whereas the overexpression of NRF2 improves sleep patterns by reducing oxidative stress (Spiers et al. 2019). Sleep fragmentation also induces NRF2 in the fly heads, along with ER-chaperones, suggesting the need to upregulate neuroprotective pathways during sleep fragmentation (Williams et al. 2016). Although many studies have failed to measure NRF2 expression after sleep deprivation, most available data from systematically reviewed animal studies suggest an increased expression of key antioxidant enzymes regulated by NRF2, such as GSH, GPx, CAT, and SOD (Villafuerte et al. 2015; Neculicioiu et al. 2023). Recently, Shah and colleagues revealed a new mechanism that explains how the antioxidant response is compromized during sleep deprivation-induced oxidative stress. Using RNA-seq and protein interaction analyses, they found that endothelial cells harvested  from healthy women, both before and after sleep restriction, exhibited a decrease in the expression of cullin neddylation-1 domain containing 3 (DCUN1D3). This protein is key in NRF2 responses and Cul3 binding. Moreover, the reduction in DCUN1D3 expression leads to an impaired antioxidant response in sleep-deprived individuals, thereby increasing their risk of cardiovascular diseases (Shah et al. 2023). In the same cohort of healthy female persons, insufficient sleep activates NF-κB in endothelial cells, suggesting a link between increased oxidative stress and inflammation after sleep restriction (Shah et al. 2022). These findings are consistent with several studies that have investigated the interconnected nature of NRF2 and NF-κB pathways. Although many important aspects of this interaction are yet to be defined, the activity of NRF2 and NF-κB is influenced by redox-sensitive factors. When NRF2 is downregulated, oxidative stress increases, which in turn amplifies cytokine production because NF-κB becomes more easily activated in oxidative conditions. Moreover, HO-1, an NRF2 target gene, seems to be at the core of the link between NRF2 and NF-κB. It has been reported that HO-1 inhibits the expression of proinflammatory genes via a mechanism that is associated with the inhibition of NF-κB activation (Soares et al. 2004; Wardyn et al. 2015; Saha et al. 2020).

As mentioned, disruption of the circadian system is thought to play a critical role in the development of sleep disturbances. NRF2 appears to be a key mechanistic link between circadian oscillations in redox balance and clock gene expression rhythms. Circadian misalignment induced by disrupted light–dark cycles or genetic loss of NRF2 contributes to ROS accumulation and impaired activity of NRF2 and its target genes, including GCLC, GCLM, and Prdx3 (Lee et al. 2013; Wible et al. 2018). Numerous studies have suggested that dysregulation of the circadian system may compromise NRF2 and the GSH system, potentially contributing to sleep dysfunction in various neuropsychiatric disorders and cancer (Morris et al. 2018; Bevinakoppamath et al. 2021). The interaction between astrocytes and neurons, essential for iron metabolism and neuronal protection from ferroptosis, depends on NRF2 activity and is crucial for proper circadian regulation. Dysregulation of interactions between astrocytes and neurons, coupled with inadequate activation of NRF2 in astrocytes, may lead to ferroptosis in neurons, particularly in dopaminergic neurons, and contribute to the disruption of circadian rhythms associated with sleep disturbances (Ishii et al. 2019; Li et al. 2023a). Dopamine metabolism, a potential cause of oxidative stress, regulates circadian rhythms and sleep transitions (Korshunov et al. 2017; Hasegawa et al. 2022). As it produces o-quinones, which are reactive neurotoxic products, dopaminergic neurons are particularly susceptible to oxidative stress and mitochondrial dysfunction. NRF2 may reduce neuronal dopaminergic oxidative stress by providing NADPH for the activity of another NRF2 target, NQO1, which maintains dopamine in the hydroquinone conformation, a less reactive intermediate in dopamine metabolism (Zafar et al. 2006; Parga et al. 2018). In the section below, we provide an overview of available evidence of how targeting NRF2 and modulating its activity may represent a viable approach to ameliorate oxidative stress linked to sleep disturbances.

## Modulation of NRF2 in Sleep Disruption

Although NRF2 deficiency may be not the primary cause of sleep disturbances, targeting its activity could represent a viable approach to improve homeostatic sleep regulation and reduce health problems associated with sleep abnormalities. A number of molecules have been used to modulate NRF2 in animal models of sleep loss (Table 2), providing proof-of-concept studies to translate in a clinical setting. However, one of the challenges is to identify NRF2 activators that exhibit a good pharmacokinetic profile and cross the blood–brain barrier (BBB). The current model is that most of these compounds induce conformational changes in KEAP1, leading to the inhibition of NRF2 ubiquitylation via dissociation of the inhibitory complex. The disruption of the ubiquitination of NRF2 allows the escape from the proteasomal degradation, activating the NRF2 transcriptional program (Dinkova-Kostova et al. 2017).

**Table 2:** Effects of NRF2 inducers in sleep deprivation models

Compounds	Models	Effects through NRF2 activation	References
Sulforaphane	Obstructive sleep apnea	•Modulation of endoplasmic reticulum (ER) stress •Protection of neurons from apoptosis •Reduction of the inflammatory response •Alleviation of cognitive impairment	Qiu et al. (2023)
Ellagic acid	Sleep deprivation	•Reduction of inflammatory response •Reduction of oxidative stress •Protection against memory impairment and anxiety in sleep-deprived mice	Wang et al. (2020a)
Corilagin	Sleep deprivation	•Inhibition of NADPH oxidase 2 (NOX2) levels •Reduction of malondialdehyde (MDA) levels •Restoration of glutathione peroxidase (GPx) and superoxide dismutase (SOD) activities	Wang et al. (2020b)
Isoflavones	Chronic sleep deprivation	•Reduction of neuroinflammation •Restoration of SOD activities •Reduction of MDA levels	Lu et al. (2022)
Melatonin	Acute sleep deprivation	•Amelioration of memory loss •Reduction of hippocampal ferroptosis •Reduction of nuclear factor-κB (NF-κB) activation •Reduction of oxidative stress	Negi et al. (2011) and Wang et al. (2021)
Farnesol	Chronic sleep deprivation	•Activation of sirtuin 1 (SIRT1) •Activation of heme oxygenase-1 (HO-1) •Restoration of GPx activities	Li et al. (2023b)
Ketones	Chronic sleep deprivation	•Inhibition of ferroptosis •Reduction of lipid peroxidation •Improvement in neuronal repair ability •Activation of SIRT1	Yang et al. (2022)
Butylphthalide	Sleep deprivation	•Reduction of inflammatory response •Inhibition of neuronal apoptosis •Amelioration of cognitive decline	Chen et al. (2022a)
Minocycline	Sleep deprivation	•Prevention of microglial activation •Alleviation of depressive-like behavior •Alleviation of anxiety	Ahmed et al. (2021)

One of the most successful case of an NRF2 activator is sulforaphane, a sulfur-rich dietary phytochemical that has neuroprotective effects (Uddin et al. 2020). In a mouse model of chronic IH and sleep fragmentation to simulate the pathological characteristics of OSA, activation of NRF2 by sulforaphane modulates ER stress, protects hippocampal neurons from apoptosis, and reduces the inflammatory response by regulating the expression of the antioxidant gene Prdx1. Overall, these results suggest that NRF2 activation may alleviate cognitive impairment induced by chronic IH and sleep fragmentation (Qiu et al. 2023). A different study with ellagic acid also suggested that activating NRF2 could potentially alleviate cognitive decline resulting from lack of sleep. Ellagic acid, a polyphenolic compound found in various plants, has been shown to protect mice from memory loss and anxiety caused by sleep deprivation. This compound reduced the inflammatory response and oxidative stress caused by sleep deprivation through the inhibition of Toll-like receptor 4 (TLR4) and activation of the NRF2/HO-1 pathway (Wang et al. 2020a). Corilagin, an ellagitannin that represents a derivative of ellagic acid, inhibited NADPH oxidase 2 (NOX2) which increases sleep deprivation-induced oxidative stress. Corilagin also normalized the elevated MDA level and the reduced activity of GPx and SOD by activating NRF2/HO-1 signaling in hippocampal tissues of sleep-deprived animals (Wang et al. 2020b).

The crosstalk between NRF2 and NF-κB signaling pathways has been demonstrated after the administration of isoflavones in a mouse model of chronic sleep deprivation. This family of phytoestrogens improved the cognitive performance of sleep-deprived animals by increasing SOD activities, decreasing MDA levels, and promoting the expression of NRF2 and its downstream targets, including HO-1 and NQO1. At the same time, isoflavones suppressed NF-κB, NOS, and COX-2, as well as the pro-inflammatory cytokine release (e.g., TNF-α, IL-6, and IL-1β) in the hippocampus of sleep-deprived mice. These results provide insight into the potential of isoflavones in alleviating oxidative stress and suppressing neuroinflammation induced by chronic sleep deprivation (Lu et al. 2022).

Melatonin, a widely recognized hormone responsible for regulating the sleep–wake cycle, acts as a powerful natural antioxidant. It also triggers the production of important antioxidant enzymes, such as SOD and GPx. Melatonin may improve circadian rhythms and sleep disorders by activating the MT1 and MT2 receptors, which are high-affinity G protein-coupled receptors involved in regulating sleep and circadian rhythms. (Liu et al. 2016; Zhao et al. 2018). Several studies have shown that exogenous melatonin can improve memory loss and hippocampal ferroptosis resulting from acute sleep deprivation by interacting with the MT2 receptor to trigger NRF2 signaling. Additionally, melatonin can regulate neuroinflammation by reducing NF-κB activation and enhancing NRF2 expression to reduce oxidative stress (Negi et al. 2011; Wang et al. 2021). SIRT1, a deacetylase enzyme, plays a key role in regulating the central circadian rhythm in the SCN by promoting the transcription of the main circadian regulators, BMAL1 and CLOCK. These circadian regulators are also regulated by melatonin to alleviate oxidative stress and attenuate cognitive impairment after sleep deprivation (Chang and Guarente 2013; Hu et al. 2023). Farnesol, a natural sesquiterpenoid, exerts neuroprotective effects against sleep deprivation-induced cognitive impairment by activating the SIRT1/NRF2 signaling pathway. Administration of farnesol after chronic sleep deprivation also increases the expressions of HO-1 and GPx in the hippocampi (Li et al. 2023b). Another study indicated that a ketogenic diet, which results in elevated levels of ketones (e.g., β-hydroxybutyrate) in the blood, could prevent sleep deprivation-induced Alzheimer’s disease (AD) by inhibiting ferroptosis, and lipid peroxidation, and improving the neuronal repair ability via SIRT1/NRF2 signaling pathway (Yang et al. 2022).

The neuroprotective effects of butylphthalide, one of the chemical constituents of celery seed, have been subject of numerous clinical trials evaluating its efficacy and safety in the treatment of various neurologic conditions (Lv et al. 2022; Wang et al. 2023). A recent study indicated that butylphthalide may be a potential candidate to alleviate cognitive deficits induced by sleep deprivation. Indeed, it has been found that butylphthalide ameliorates sleep deprivation-induced cognitive decline by reducing the inflammatory response and inhibiting neuronal apoptosis. These effects were associated with the modulation of the NRF2/HO-1 pathway (Chen et al. 2022a). Based on its powerful anti-inflammatory and neuroprotective activities, minocycline, a tetracycline antibiotic that can cross the BBB, is a candidate for the treatment of depression and anxiety. Moreover, minocycline has been reported to exert its anti-inflammatory actions by modulating microglial activation through SIRT1 activation (Soczynska et al. 2012; Wu et al. 2020). Sleep-deprived animals exhibit activated microglia (i.e., neuroinflammation) and decreased levels of NRF2 in the hippocampus. However, treatment with minocycline prevented microglial activation, restored NRF2 levels, and alleviated depressive-like and anxiety-like behavior after sleep deprivation (Ahmed et al. 2021).

## Conclusions

Identifying the key regulators of sleep is essential to understanding the detrimental effects on health associated with sleep disruptions. Although the physiological and health implications of the connection between sleep and oxidative stress have started to be revealed, the restorative function of sleep in mitigating oxidative stress in the brain and peripheral tissues remains elusive. Consequently, further research is needed to identify precisely how ROS and RNS regulate sleep behavior. It is noteworthy that these reactive species, at low physiological levels, may be involved in the homeostatic control of sleep. Likewise, recent experimental evidence in various sleep disturbances supports the idea that cellular accumulation of ROS and RNS contributes to poor sleep quality, sleep disorders, and circadian rhythm disruptions. Therefore, even though it is experimentally challenging, it is crucial to induce varying levels of ROS and RNS, optimizing the dose and duration of the treatments to determine the specific effects of these reactive species on sleep patterns and to identify any alterations in sleep-related pathways.

Given that these species can accumulate not only through increased production but also through decreased clearance, an inadequate antioxidant response, particularly associated with a dysregulation of NRF2 transcriptional activity, could explain their gradual accumulation. Accordingly, oxidative and nitrosative stress may contribute to the emergence of sleep disturbances, fueling a pathophysiological loop with neuroinflammation induced by these abnormalities. Although NRF2 has not yet been fully investigated in the context of sleep, this multifunctional transcription factor may significantly influence sleep quality. It may induce a large number of genes involved in the regulation of sleep, including those regulating redox homeostasis and inflammation pathways. Current studies have revealed substantial dysregulation of NRF2 in various sleep abnormalities, particularly in models of sleep deprivation, thereby offering novel insights into the underlying physiology of sleep and its disturbances. In addition, some of these studies have explored the use of compounds targeting the NRF2 system to reinforce its activity during sleep disturbances. Taking together all the studies discussed in this review, it seems reasonable to conclude that NRF2 is part of the sleep biochemical regulatory network. Given that NRF2 regulates the expression of numerous genes associated with oxidative stress and inflammation, the application of omics methodologies, which allow for the simultaneous measurement of hundreds to thousands of molecular targets, may help to fully delineate how NRF2 activity influences the complex interplay between redox homeostasis and sleep regulation. These studies should lead to a greater understanding of the role of NRF2 in the redox balance of sleep and its homeostatic regulation.

## Data Availability

Enquiries about data availability should be directed to the authors.
